# Reducing Falls in Hospitalized Children and Adolescents with Cancer and Blood Disorders: A Quality Improvement Journey

**DOI:** 10.1097/pq9.0000000000000755

**Published:** 2024-08-30

**Authors:** Lisa K. Morrissey, Phuc Ho, Maya Ilowite, David A. Johnson, Colleen M. Nixon, Marissa K. Thomas, Julie A. Waitt, Amy Wierzchowski, Ashley M. Renaud

**Affiliations:** From the * Department of Nursing, Hematology/Oncology/Stem Transplant Program, Boston Children’s Hospital, Boston, Mass.; † Division of Hematology/Oncology, Dana-Farber Boston Children’s Cancer and Blood Disorders Program, Department of Pediatrics, Harvard Medical School, Boston, Mass.; ‡ Department of Pediatrics, Boston Children’s Hospital, Boston, Mass.; § Department of Physical Therapy; Boston Children’s Hospital, 300 Longwood Avenue, Boston, Mass.; ¶ Clinical Education, Informatics, Quality & Practice, Boston Children’s Hospital, Boston, Mass.

## Abstract

**Background:**

Falls in hospitalized pediatric patients represent a serious patient safety concern. Children and adolescents with cancer and blood disorders have inherent risk factors that increase the likelihood of injury from falls. The Hematology/Oncology (HO) and Stem Cell Transplant (SCT) inpatient units at Boston Children’s Hospital embarked on a multiyear quality improvement journey to reduce inpatient falls in this population.

**Methods:**

A targeted Falls Reduction Task Force implemented key initiatives between 2020 and 2023. These include enhancing communication strategies to heighten awareness of the highest fall-risk patients, conducting a formal apparent cause analysis on every fall with injury, and initiating a physical therapy-led program to reduce deconditioning. Outcome measures were total falls, rate of preventable falls with injury per 1000 patient days, and days between preventable falls with injury. Our quality improvement team used statistical process control charts to track changes over time.

**Results:**

The combined rate of preventable falls with injury per 1000 patient days decreased from 0.63 in fiscal year (FY) 2020 to 0.25 in 2023. The SCT and HO units achieved a maximum of 442 days and 410 days, respectively, between preventable falls with injury in 2021–2023, compared with 124 and 117 days in 2020. The two units observed a 51% reduction in total falls over 4 years.

**Conclusions:**

A multifaceted fall reduction quality initiative effectively reduced preventable falls with injury on pediatric HO and SCT inpatient units, thereby reducing avoidable harm in a vulnerable patient population.

## Introduction

Falls pose a serious safety risk to patients in acute care hospitals, frequently prolonging or complicating inpatient stays. Extensive literature is available on adult patient falls, yet evidence-based data on the prevention of pediatric inpatient falls remains sparse.^1^ Falls are a leading cause of unintentional and preventable injury in hospitalized children.^2^ The estimated rate of inpatient falls in children is lower than in adults, yet the incidence of injury is significant.^3^ There is a critical gap in the literature on the etiology of pediatric falls and effective fall prevention strategies in children with complex medical needs.^4^

The Joint Commission requires organizations to reduce the risk of a patient falling and the harm resulting from falls.^5^ Falls are a nurse-sensitive indicator tracked by Magnet designated institutions.^6^ The Children’s Hospitals’ Solutions for Patient Safety created a pediatric falls bundle that includes four standard elements: screen patients for risk of falls, identify and communicate patients at risk for falls and injury, ensure a safe environment, and review of safety protocols with patients and caregivers.^7^

Leadership engagement and a multidisciplinary team approach to eliminating harm are essential at the institutional level. Meaningful impact requires content expertise with authority to execute real-time changes guided by quality experts. Fall reduction initiatives must be integrated into the hospital safety program, with a formal leadership review of progress measured via dashboard metrics.^8,9^

### Prevalence and Incidence

National benchmarking of pediatric falls did not begin until 2013, mainly due to variability in the definition of pediatric falls, risk factors, and effective prevention strategies.^1^ The estimated fall rate for hospitalized patients ranges from 0.5 to 2.20 per 1000 patient days.^3,7,10,11^ A staggering 61% of all falls are anticipated physiologic falls, which could be prevented.^12^ The Centers for Medicare & Medicaid Services (CMS) do not reimburse costs for inpatient falls-related injuries.^13^

### Risk Assessment

Hospitalized children are in an unfamiliar environment, increasing the risk of falling regardless of age or diagnosis. The child’s medical condition, treatments, and medications further compromise normal developmental and prospective control.^3^ Implementing a validated risk assessment tool is the most cited intervention to reduce falls in pediatric hospitals, suggesting that clinician awareness is an effective strategy in reducing falls.^2,3,10,14,15^ Fall-risk screening tools created for adults do not have a predictive value when extended to the pediatric population.^16^ It remains unclear which pediatric fall-risk assessment tools most accurately assess pediatric fall risk.^1,15^ Prevention measures that enhance patient and caregiver participation through visual tools have been published, such as educational posters and standardized programs to reduce drops and falls in hospitalized infants.^17,18^ Interventions that specifically address pediatric falls in high-risk populations are limited.^12^

At Boston Children’s Hospital (BCH), infants to young adults with cancer and blood disorders are cared for on the 30-bed HO unit and the 14-bed SCT unit. In FY 2023, the average daily census was 26.8 on the HO unit and 12.6 on the SCT unit, with an average length of stay (ALOS) of 7.61 days on the HO unit and 14.69 days on the SCT unit. ALOS for a stem cell transplant admission was 26.5 days.

Our hospital uses the General Risk Assessment for Pediatric Inpatient Falls (GRAF PIF) predictor model as a fall-risk assessment tool; variables include length of stay >5 days, need for physical/occupational therapy, use of seizure medications, absence of IV/heparin lock, and orthopedic disorders.^3,19^ Patients on the HO and SCT units are frequently screen as high fall risk using GRAF-PIF due to the length of stay. Patients with impaired bone marrow function often require preventative interventions after a fall, such as a platelet transfusion, to minimize bleeding risk, elevating the fall severity rating. A study of pediatric inpatients found that children with bleeding precautions and blood disorders are four times as likely to fall while hospitalized as children without these conditions. Bone fragility and a history of falls were also cited as fall predictors.^15^

The National Database of Nursing Quality Indicators (NDNQI) compared falls among seventy-three participating pediatric medical units between 2017 and 2019. Our HO unit observed a higher rate of total falls than the consortium average in seven of eight quarters. In response, an HO/SCT Fall Reduction Task Force (FRTF) formed in collaboration with our Department of Medicine Quality Improvement (QI) team to conduct an in-depth review of factors contributing to falls. We established a goal to decrease inpatient falls with injury by 20% annually by implementing key interventions guided by QI methodology.

## METHODS

**Fig. 1.**
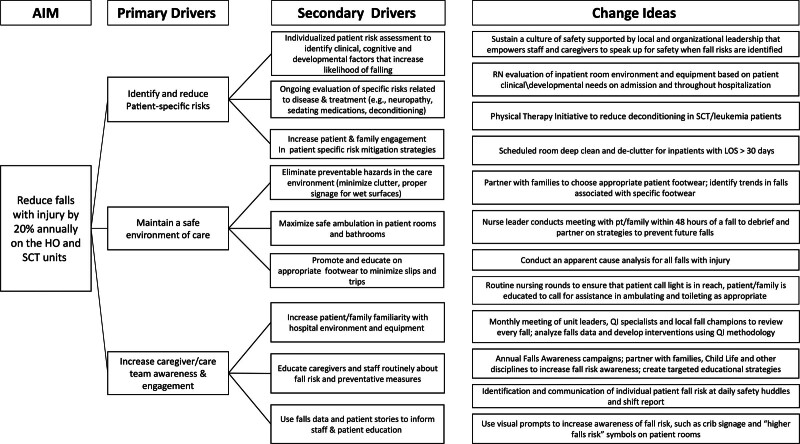
Key Driver Diagram. The KDD allowed the Falls Reduction Task Force to identify specific risk factors contributing to falls on the HO/SCT units. The group defined the primary drivers as patient-specific risks, caregiver/care team engagement, and maintaining a safe care environment. Our team identified secondary drivers related to each category. This exercise guided targeted interventions, identified as “Change Ideas.”

The HO/SCT FRTF consists of subject matter experts, including staff nurses, nursing directors, a nursing professional development specialist, QI specialists, and a program-based physical therapist. The QI specialist led the team in a key driver diagram (KDD) exercise to identify the specific risk factors contributing to falls on the HO/SCT units. The group classified risk factors in relationship to one of three categories: patient-specific risks, environment of care, and care team/caregiver engagement, consistent with themes from a previous multisite study.^20^ The KDD illustrated that multiple factors contribute to falls and that patient-specific risk factors differ by age, diagnosis, mobility status, disease and treatment complications, the inpatient room environment, care team engagement and caregivers’ presence (or absence) at the bedside (Fig. 1).

We describe key interventions implemented by the task force, including the commitment to conduct an apparent cause analysis (ACA) on every fall with injury, enhanced techniques to improve communication of fall risks to staff and families, and initiation of a physical therapy (PT) program to reduce deconditioning in patients with long hospitalizations.

### Falls SME/QI Collaboration

Our QI specialist reviews the unit metrics at the monthly FRTF meeting, including total falls and rate of patient falls with injury per 1000 patient days, days between falls, and falls by category. The SMEs review ACAs conducted in the previous month to understand the circumstances around each fall and discuss patient-specific prevention strategies. The members representing the HO/SCT program at the institution-wide Falls Committee monthly meeting report on organizational data and findings.

### ACA Reports

An ACA is a QI process by which local stakeholders conduct a limited investigation of an event to address the immediate problem and collect information that identifies trends within the setting.^21^ At BCH, ACAs are performed by unit and program leaders to identify meaningful action items for their areas and to inform system-wide recommendations for leaders within the organization. Nurse champions who received training on the ACA process now initiate an ACA for all falls with injury on the HO and SCT units, using a standardized guided tool. The ACA reports are reviewed at multiple program meetings as an opportunity for education and potential practice changes derived from actual patient scenarios.

### Enhancing Caregiver Engagement after a Patient Fall

Patients with a recent history of falls are at higher risk for future falls.^13,20^ An inpatient nurse leader from the unit meets with the patient and caregivers within 24-48 hours of a fall to review the event, reinforce prevention strategies, and discuss patient-specific risk factors such as developmental age, medication side effects, and treatment complications. These discussions often led to valuable insights. Caregivers point out vulnerabilities in the patient’s environment, such as slip hazards or inconsistencies in the inpatient room layout. Patients and families often share a lack of awareness of the increased risk of falling while hospitalized and have voiced a reluctance to “interrupt” staff for assistance with ambulation and toileting. This meeting is ideal for reeducating and empowering patients and caregivers to partner in fall prevention strategies.

### Higher Fall-Risk Category/Signage

During twice-daily multidisciplinary safety huddles, the charge nurse announces high fall-risk patients by name and specific risk factors. The bedside nurse places a yellow magnet on the patient/team whiteboard in the nursing conference room and outside each room, indicating high fall-risk patients. RNs communicate patient fall risk twice daily at the change of shift report.

### SCT PT Pilot

Historically, SCT patients at our institution received a PT consultation on admission and education on a daily exercise program. The clinical team often did not identify or address deconditioning until late hospitalization. A literature review to determine best practice in PT intervention during SCT revealed that optimal outcomes were observed when SCT patients were treated at a frequency of three to five times per week with interventions to address strengthening, flexibility and endurance.^22–24^ A PT pilot was developed and implemented in July 2020, coinciding with a drop in hospital census during the COVID-19 pandemic, which increased PT services. The PT department dedicated a team of four to the SCT population for the pilot. SCT patients were seen thrice weekly, beginning with a pre-SCT evaluation followed by initiation of strengthening, balance, and endurance interventions. Physical therapists conduct the Pediatric Balance Scale and the 30-second sit-to-stand test at initial evaluation, during regular intervals during hospitalization and predischarge to monitor balance and functional strength. If a patient experienced a decline in function during admission, the frequency increased to five to seven times per week based on our inpatient PT frequency protocol. The physical therapists developed accommodations for patients who refused PT at this frequency.

The PT team leader conducted a chart review to compare PT interventions in twenty-two patients aged 5–18 years admitted for HSCT in the 6-month preintervention period (January–June 2020) to twenty-seven patients who met the same criteria in the 6-month postintervention period (August 2020–January 2021). The number of successful PT sessions in the first 28 days of hospitalization increased to 8.68 versus 4.37 per patient preintervention, representing a 50.3% increase in successful interventions during the first 28 days of transplant. When the data on the reduction in falls indicated success, PT leadership approved maintaining the staff of four PTs on the SCT unit to accommodate the increase in patient appointments.

The PT pilot expanded to the HO unit to include patients newly diagnosed with acute lymphoblastic leukemia and acute myelogenous leukemia. Historically, leukemia patients received PT consults only if the clinical team identified a specific need or an existing deficit. We modified the oncology admission order set to include a PT consult to ensure that the evaluation will occur within 5 days of admission. The physical therapist develops a care plan consisting of strengthening, balance, and endurance-based activities with ongoing strength and sensory deficits monitoring.

The PT pilot invited collaboration from other disciplines. Child Life Services promoted mobility outside of PT sessions by creating activities encouraging ambulation, such as scavenger hunts in the hallway. PT partners with Occupational Therapy, requesting consults when indicated, to provide recommendations for activities of daily living, such as bathing and toileting. The inpatient teams support the PT initiative by reinforcing patient and parent education on the risk of muscle atrophy with decreased activity.

### Measurement

The hospital-wide Fall Prevention Committee, composed of subject matter experts from local units, meets monthly to classify all inpatient falls into one of eight categories based on the NDNQI definitions, which include health status, developmental, play, environmental, misjudgment, attentiveness, modesty, and staff assisted.^25^ Organizations submitting data to NDNQI receive fall-related definitions and categories to ensure data consistency. Based on standard definitions, falls are classified as preventable, possibly preventable, or not preventable. The BCH Falls Committee conducts an adjudication process through discussion and expert consensus to determine a fall’s category, severity rating and preventability. Our QI team organizes the Oncology/SCT-specific data and compares falls by category using Pareto charts. Our team incorporated metrics associated with falls into the HO/SCT quality dashboard, which program leaders review at our monthly QI meeting.

## RESULTS

**Fig. 2.**
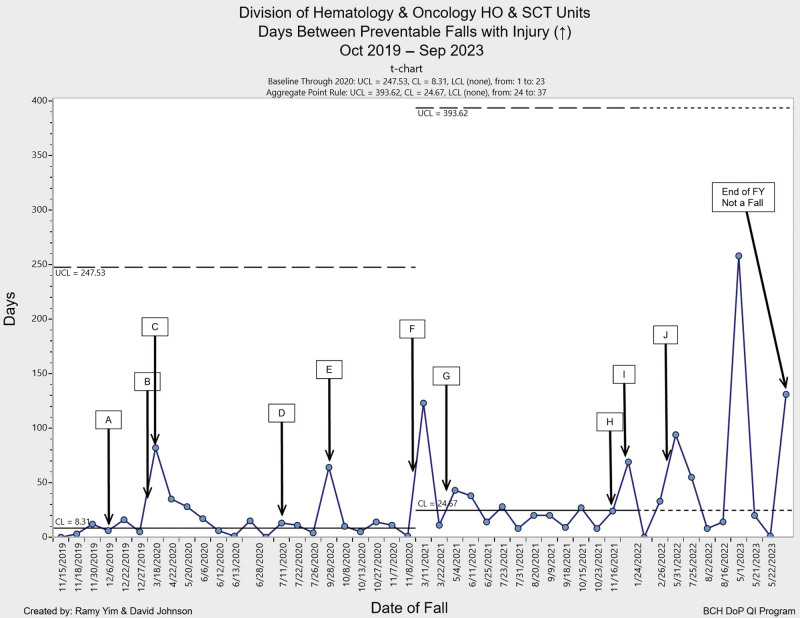
Days between preventable falls with injury t-chart: The mean days between preventable falls with injury increased to 24.67 days in FY 2021, compared with 8.31 days in the baseline period. Key interventions are annotated in Figure 2 and Figure 3 as follows: A—Falls Reduction Task Force formed (December 19), B—ACA, All Falls with Injury (Jan 2020), C—COVID-19 Pandemic Begins (Mar 20), D—PT Pilot Program on SCT Unit (Jul 2020), E—Patient/family meeting postfall (Sep 2020), F—Expansion of PT Pilot to leukemia patients (Dec 2020), G—Pediatric cardiac chairs on units (Apr 2021), H—New Socks Program on SCT Unit (Dec 2021), I—New Socks Program on HO Unit (Jan 2022), J—Crib safety signage implemented (Mar 2022)

**Fig. 3.**
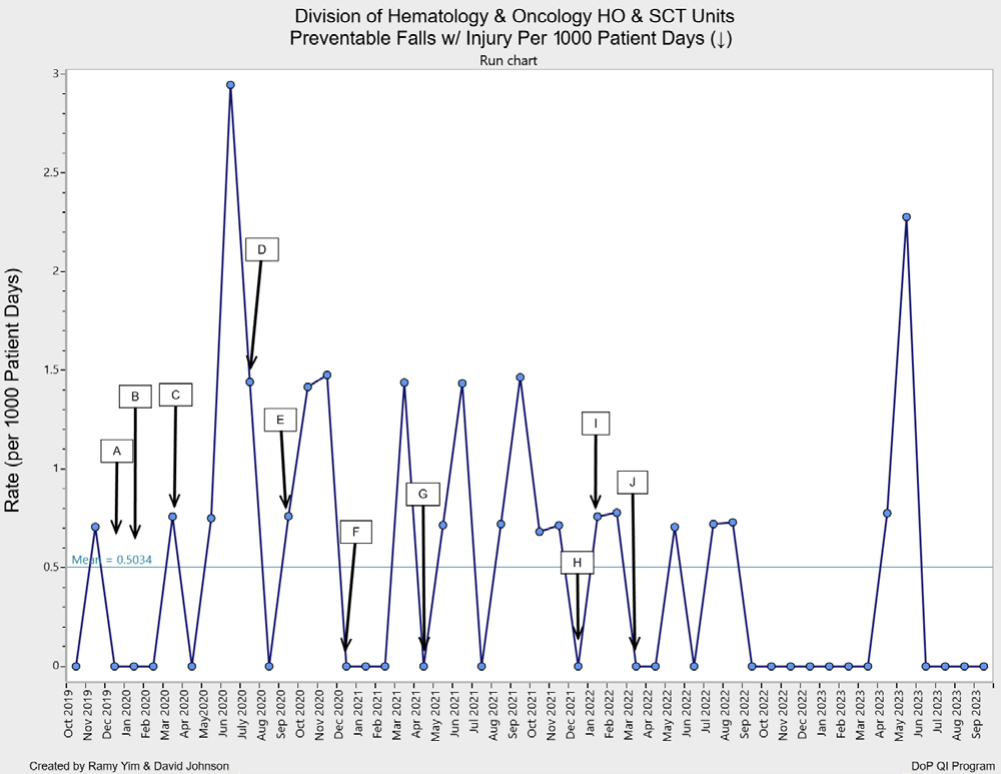
Preventable falls with injury rate run chart. The preventable falls with injury per 1000 patient days decreased to 0.25 in FY 2023, compared with 0.63 in 2020, 0.74 in 2021 and 0.42 in 2022. We referenced the same interventions annotated in Figure 2.

**Fig. 4.**
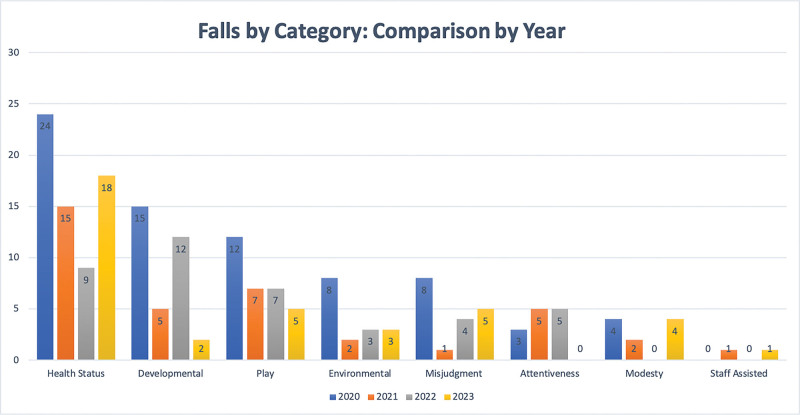
Annual comparison of falls by category. Based on NDNQI definitions, our team classifies inpatient falls into one of eight categories. Our QI team organizes the H/O and SCT falls by category into Pareto charts. This graph compares the falls annually by category over the 4-year intervention period.

Following the implementation of these interventions, the HO and SCT units observed a notable decline in total falls and preventable falls with injury, as well as an increase in days between falls. The combined unit data showed an increase to 24.7 mean days between preventable falls with injury in 2021 compared with 8.3 days in 2020 (Fig. 2). We achieved a maximum of 263 days with no preventable falls with injury in the postintervention period, with the SCT unit reaching a maximum of 442 days and the HO unit a maximum of 410 days, compared with 124 and 117 days, respectively, in the preintervention period. The combined rate of preventable falls with injury per 1000 patient days on the two units decreased to 0.25 in 2023 compared with 0.63 in 2019, 0.74 in 2021 and 0.42 in 2022 (Fig. 3). Pareto charts revealed that health status was the top category for HO/SCT patient falls, followed by developmental falls, falls during play and falls related to the patient environment. Falls decreased in all four categories in 2023 compared with 2020 (Fig. 4). The total number of falls decreased by 51% when comparing 2023 to 2020.

## DISCUSSION

This report describes a multi-year journey in the HO and SCT units at a freestanding academic pediatric hospital to address the refractory problem of inpatient falls. Our findings illustrate that building a culture of fall safety and awareness impacts a preventable patient injury risk. Not all pediatric patients have equal risks; the etiology of falls varies depending on factors, including age, health status, caregiver engagement, and the physical environment of inpatient rooms and care areas. Identification and communication of patient-specific risks are key to prevention. For example, hospitalized infants and toddlers are at the highest risk for falls from a crib or raised surface and developmental falls while ambulating or playing. Adolescents struggling to maintain autonomy and privacy may be reticent to assistance with toileting. Recognition that patients and caregivers require age and risk-specific targeted education is a key step toward fall prevention.

A PT initiative to reduce deconditioning led to a significant decrease in falls related to health status, the category associated with most injury falls. Patients undergoing stem cell transplants have a predictably long hospitalization and are highly prone to decreased strength due to muscle atrophy. The PT team successfully expanded the deconditioning reduction initiative to leukemia patients who also experience prolonged hospitalizations.

Consistent verbal and visual safety cues promote a culture of fall-risk awareness. On our units, the charge nurse leading daily safety huddles announces the days between falls with injury and communicate the patients by name who are at the highest risk for falls. Our unit administrators post days between falls on the unit QI board daily. Our team celebrates significant milestones, such as achieving one year without falls or injuries. We have observed that engaging patients and caregivers in a postfall discussion highlights the factors that led to a fall and offers patient education to reduce the risk of a subsequent fall.

Identifying nurses, physical therapists, and QI specialists to dedicate time to this initiative required an upfront commitment of resources. Buy-in from local and hospital leadership to approve the additional resources was key, and the investment has paid off in the reduced frequency of patient falls.

Limitations include the fact that this single-center QI project focused on a specialized patient population at a pediatric hospital and may not apply to other pediatric settings or patient groups. We did not study each intervention individually to evaluate its impact on reducing falls.

## CONCLUDING SUMMARY

Pediatric falls have unique contributing factors compared with adults and represent a serious, preventable injury risk in hospitalized children and adolescents. Pediatric patients with cancer and blood disorders and those undergoing stem cell transplants are a vulnerable population who possess risk factors that are predictors of falls with injury. A multipronged, team approach to reducing fall risk in HO and SCT patients has effectively reduced the number of falls with injury and heightened staff and caregiver awareness. Further investigation will determine if our findings are generalizable to HO/SCT units in other institutions and high-risk pediatric specialty populations.

## ACKNOWLEDGMENTS

The authors thank Dr. Laura Wood, Dr. Jonathan Whiting, Jeffrey Durney and the BCH Department of Quality and Safety for the ongoing support of this initiative, and Dr. Mary Poyner-Reed for her guidance on the manuscript. We thank the nurses, clinical assistants, providers, physical therapists, support staff, and caregivers on the HO/SCT units for their commitment to patient safety and quality care.
